# Technology Acceptance in Mobile Health: Scoping Review of Definitions, Models, and Measurement

**DOI:** 10.2196/17256

**Published:** 2020-07-06

**Authors:** Camille Nadal, Corina Sas, Gavin Doherty

**Affiliations:** 1 School of Computer Science and Statistics Trinity College Dublin Dublin Ireland; 2 School of Computing and Communications Lancaster University Lancaster United Kingdom

**Keywords:** Technology Acceptance Lifecycle, patient acceptance, mobile applications, mHealth, mobile phone

## Abstract

**Background:**

Designing technologies that users will be interested in, start using, and keep using has long been a challenge. In the health domain, the question of technology acceptance is even more important, as the possible intrusiveness of technologies could lead to patients refusing to even try them. Developers and researchers must address this question not only in the design and evaluation of new health care technologies but also across the different stages of the user’s journey. Although a range of definitions for these stages exists, many researchers conflate related terms, and the field would benefit from a coherent set of definitions and associated measurement approaches.

**Objective:**

This review aims to explore how technology acceptance is interpreted and measured in mobile health (mHealth) literature. We seek to compare the treatment of acceptance in mHealth research with existing definitions and models, identify potential gaps, and contribute to the clarification of the process of technology acceptance.

**Methods:**

We searched the PubMed database for publications indexed under the Medical Subject Headings terms “Patient Acceptance of Health Care” and “Mobile Applications.” We included publications that (1) contained at least one of the terms “acceptability,” “acceptance,” “adoption,” “accept,” or “adopt”; and (2) defined the term. The final corpus included 68 relevant studies.

**Results:**

Several interpretations are associated with technology acceptance, few consistent with existing definitions. Although the literature has influenced the interpretation of the concept, usage is not homogeneous, and models are not adapted to populations with particular needs. The prevalence of measurement by custom surveys suggests a lack of standardized measurement tools.

**Conclusions:**

Definitions from the literature were published separately, which may contribute to inconsistent usage. A definition framework would bring coherence to the reporting of results, facilitating the replication and comparison of studies. We propose the Technology Acceptance Lifecycle, consolidating existing definitions, articulating the different stages of technology acceptance, and providing an explicit terminology. Our findings illustrate the need for a common definition and measurement framework and the importance of viewing technology acceptance as a staged process, with adapted measurement methods for each stage.

## Introduction

### Background

Technology acceptance is a major challenge faced by designers of new technologies. In health care, patients are a vulnerable population, and their data are considered sensitive, especially in the case of stigmatized conditions such as those concerning mental health [1,2]. As mobile technology becomes increasingly pervasive in health care [3], the expanding use of potentially invasive technologies such as sensing and machine learning is likely to lead to greater concerns among users, exacerbating existing problems with attrition, and willingness to use new technologies. In addition, health care providers’ perception of a technology is likely to affect treatment delivery, especially if it is not considered sufficiently acceptable [4].

The last decade has seen an increasing number of studies addressing this issue in the mobile health (mHealth) domain. A recent systematic review by Wozney et al [5] revealed that acceptability was the most frequently measured outcome in studies on mental health technologies for anxiety and depression. Designing for acceptance is not straightforward, as the user journey is complex and often nonlinear. Patients who decide to try an application will not necessarily use it in the long run, and similarly, someone who has stopped using a system might go back to it later [6,7]. Conditions such as depression might also impact patients’ perception of their experience with technology [8] and thus affect their acceptance. Different stages punctuate the users’ journey with technology where they, consciously or not, repeatedly make the decision to keep using or to abandon it [9]. In addition, the extent to which users have appropriated and integrated technology into their lives may impact their decision to maintain use.

#### Terminology and Definitions

An evolving terminology and range of definitions can be found in the literature discussing technology acceptance. Terms such as acceptability, acceptance, and adoption are often employed, sometimes interchangeably. For instance, Al-Youssef [10] refers to acceptability as users’ willingness to use a system while citing the definition of acceptance given in Dillon and Morris [11]. These terms are sometimes equated to other human-computer interaction (HCI) concepts such as user satisfaction [12]. Yet, a part of the literature tries to differentiate the process of technology acceptance from existing concepts. For instance, Schade and Schlag [13] describe acceptability as “a prospective judgement of measures to be introduced in the future,” which they detail as “the target group will not yet have experienced the new measures.” Close to this interpretation, Adell [14] equates acceptance to “the degree to which an individual intends to use a system and, when available, incorporates the system in his/her [driving].”

The Cambridge Dictionary defines acceptability as “the quality of being satisfactory and able to be agreed to or approved of” [15] and acceptance as a “general agreement that something is satisfactory or right” [16]. This suggests that acceptability is a *quality* of an object from the perspective of a stakeholder, whereas acceptance is more of a process relating to a stakeholder’s *interaction* with this object. In the context of HCI, it translates into a user-system dyad.

In the same line, Proctor et al [17] define acceptability as “the perception among implementation stakeholders that a given treatment, service, practice, or innovation is agreeable, palatable, or satisfactory.”

With the literature highlighting the importance of temporality in user experience research [18,19], some authors attempt to integrate a temporal dimension into the process of technology acceptance. In that respect, a group of authors supported the idea of a multistage process. Martin et al [20] and Distler et al [21] define technology acceptability as one's perception of a system before use. They distinguish acceptability from acceptance by equating acceptance to the users’ perception of the system after use. Terrade et al [22] and Garces et al [23] go a step further, stating that acceptance refers to the initial use of a system in controlled settings. Reerink-Boulanger [24] introduces the continuum acceptability-acceptance- appropriation, describing acceptability as the subjective perception of the use of a system, acceptance as the first interactions with the system and appropriation as the use of the system by an individual in interaction with other individuals (translated from the original paper in French). Other definitions of appropriation include “a process of social construction in which the actions and thoughts of the user are shaped by the technology, while the meaning and effects of the technology are shaped through the users’ actions” [25] and “the process through which technology users go beyond mere adoption to make technology their own and to embed it within their social, economic, and political practices” [26].

Whereas a consistent framework seems to emerge from these definitions, another term, adoption, comes into the picture. Some authors such as Carroll et al [27] equate the process of adoption with the entire user journey: “a multi-phase process starting with deciding to adopt (selecting, purchasing, or committing to use it) and then achieving persistent use.” In contrast, Karahanna et al [28] describe “preadoption” and “postadoption (continued use)” stages and present adoption as an event allowing progress from one stage to the other. Similarly, Rogers [29] defines adoption as the user’s decision to “make full use of an innovation as the best course of action available.”

#### Models

Another strand of work has attempted to identify the factors influencing technology acceptance, the most well-known of which is the technology acceptance model (TAM) of Davis [30]. In the health domain, Kim and Park [31] have proposed a health information technology acceptance model (HITAM). This model integrates the TAM by Davis, along with antecedents and health-related constructs (health status, health beliefs and concerns, behavioral beliefs, and perceived health threat). Other models, such as the pervasive technology acceptance model [32], integrate the influence of demographics and trust on user acceptance. Dou et al [33] introduced the constructs *relationship with doctor* and *resistance to change*. Finally, Cheung et al [34] introduced constructs related to privacy and consumer innovativeness.

However, the temporal dimension is missing from these models. This raises the question of whether acceptance is motivated by the same factors and in the same manner, regardless of how long the system has been used. As stated in a review on user engagement, describing a concept as a process, rather than a discrete state, “enables the analysis of change over time” [35]. In line with this, some authors propose models that incorporate temporality. Karapanos et al [36] showed that, in their study, qualities that satisfied participants at initial use did not necessarily motivate prolonged use. Building on this, Karapanos et al [37] explored factors influencing user experience over time; they proposed a temporal framework that identifies 4 stages: anticipation (formation of expectations before any experience of use), orientation (users’ initial experiences), incorporation (how the product becomes meaningful in the user’s life), and identification (how the product participates in users’ social interactions). Although the work addresses user experience rather than acceptance, the authors claim that different qualities contribute to user experience over time, and thus, time alters the way users experience technology. As the extent to which users accept a technology is undeniably linked to their usage experience—or lack of—it is relevant to ask how user acceptance evolves over time and how the influencing factors vary accordingly.

Further exploring the temporal dimension of acceptance, Greenhalgh et al [38] proposed a framework for nonadoption, abandonment, scale-up, spread, and sustainability (NASSS). Directed at health care technologies, the NASSS framework describes different domains influencing technology adoption. It adds a temporal dimension targeting technology’s “continuous embedding and adaptation over time.” The framework is designed to be used at different points in time (at early design, after deployment, and after abandonment), which distinguishes it from the other models.

#### Measurement

Both definitions and more detailed models of acceptance may find expression in the form of approaches to measurement. Examples of acceptance studies at different stages of the technology lifecycle can be found in the literature, in some cases employing measurement tools based on theoretical models. This includes gathering of qualitative data via focus groups and interviews based on the TAM [39], a survey based on the unified theory of acceptance and use of technology [40], and interviews based on the fast form TAM [12].

Proctor et al [17] defined eight outcomes for the measurement of implementation of health interventions, two of which were acceptability and adoption. In a recent paper, Hermes et al [41] proposed to adapt the characterization of these outcomes for behavioral intervention technologies. The authors explicitly link the adoption outcome to the use (or intention to use) of a system. In addition, they state that “usability clearly overlaps with acceptability”, and thus, usability measurement tools (such as the system usability scale) could be used to assess acceptability. It would be interesting to see whether researchers chose to measure a system’s usability to make inferences regarding its acceptability.

Although there seems to be an effort in the research community to ground assessment methods in existing theoretical frameworks, custom measurement tools such as ad hoc surveys are also used to investigate technology acceptance (eg, Allen and Seaman [42]). The use of ad hoc tools allows explorations of acceptance to be tailored to a particular context but makes it more difficult to compare results across projects.

### Objective

A number of definitions and models of technology acceptance are available in the literature, several of which make important distinctions between the different stages of the process. Omitting that distinction in the terminology, and using the terms acceptability, acceptance, and adoption interchangeably can create ambiguities about what is actually being measured, making the replication of interventions and comparison of results difficult. Such ambiguity is highlighted in a review [4] that showed the confusion around the concept of technology acceptability in the health care literature. Furthermore, a review of definitions for electronic health [43] stressed the importance of common terminology for interdisciplinary collaboration. Technology acceptance is a particularly significant challenge for the design of mHealth care technologies; hence we focus on that particular context. In this paper, we present the results of a scoping review of the mHealth literature addressing the following questions:

- How do researchers define technology acceptance?
- What terminology is used to refer to technology acceptance?
- How do researchers measure technology acceptance?
- How do researchers make use of existing models of acceptance?

In addition to outlining research practices to evaluate acceptance, this review reveals the potential limitations and areas for improvement of existing models. A better understanding of these elements contributes toward the development (or improvement) of methodologies and measurement tools for addressing technology acceptance within the development of mHealth applications. Informed by this analysis, a further contribution is made by integrating and disambiguating existing definitions. To this end, we present a lifecycle of the process of acceptance, providing researchers with a common terminology to report results, and help them to measure the evolution of user acceptance over time.

## Methods

We performed a scoping review to map relevant literature in the field of mHealth. In contrast to systematic and narrative reviews [44], scoping reviews allow for a broad but structured exploration that permitted us to examine the range of definitions and measurements of technology acceptance, and to identify gaps and inconsistencies in the existing literature. We followed the framework developed by Arksey and O'Malley [45], which consists of the stages outlined below.

### Identifying the Research Question

We were interested in how technology acceptance was understood, measured, and reported in mHealth studies.

### Searching for Relevant Studies

We searched the PubMed database for papers addressing technology acceptance. Following preliminary searches to assess the relevance of search criteria, a search of the Medical Subject Headings (MeSH) terms “Patient Acceptance of Health Care” and “Mobile Applications,” without time restriction, resulted in a corpus of 287 articles starting from 2013.

### Selecting Studies to Include

**Figure 1:**
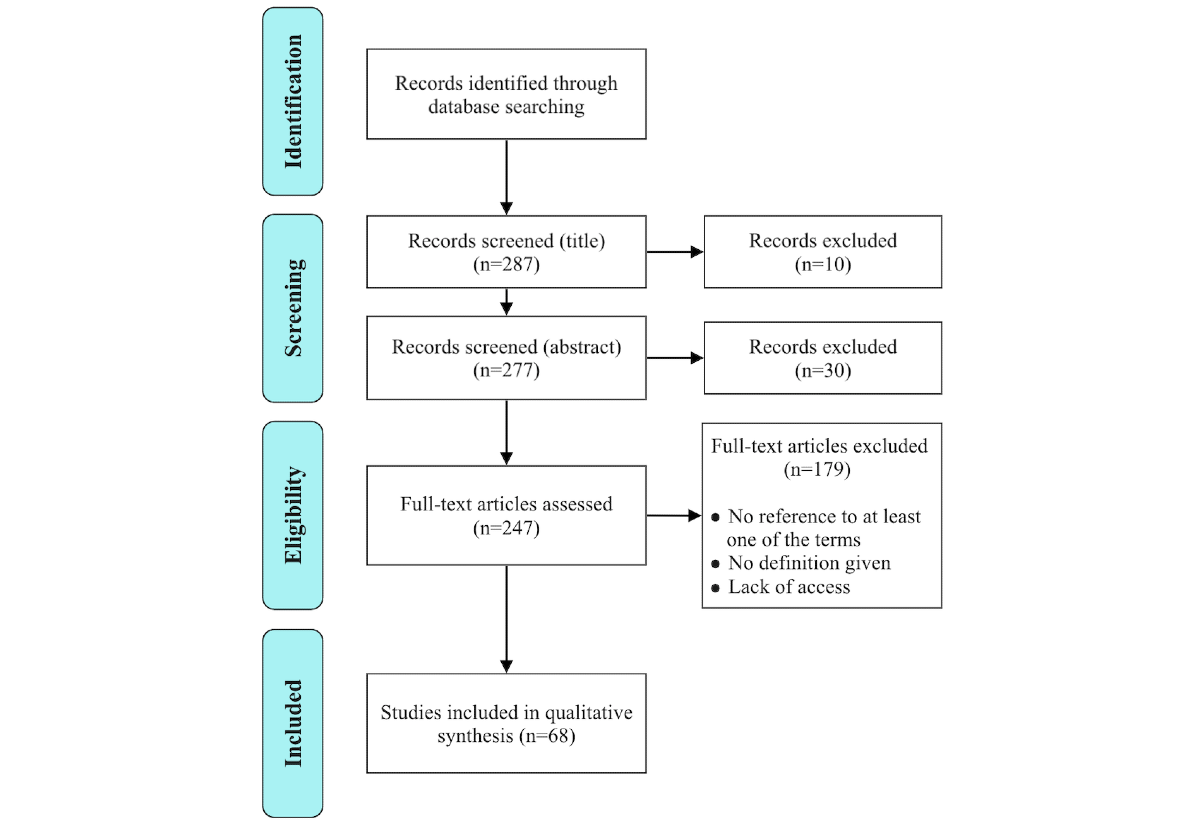
Flow diagram.

Inclusion criteria were subject to discussion between the 3 authors; 2 random samples of 10 papers were independently assessed for inclusion by 2 pairs of authors (first and second, first and third). Disagreements concerning the definitions of inclusion criteria were resolved by explicitly stating these criteria as following: a publication was considered relevant if (1) it contained at least one of the terms “acceptability,” “acceptance,” “adoption,” “accept,” or “adopt”; and (2) defined the concept in question with (1) a full definition; (2) a synonym; or (3) an operationalized definition (ie, means used to measure the concept). Some articles were associated with the specified MeSH terms but did not directly discuss technology acceptance. Articles were excluded if they did not contain any of the 5 terms above, did not provide a definition, or if their web version was not accessible. Among those, 40 were excluded at the screening stage (Figure 1) because they did not address acceptance in the sense of technology acceptance (eg, Acceptance and Commitment Therapy). A total of 68 relevant publications were included in the review.

### Charting the Data

The codes used for analysis aimed to expose how technology acceptance was referred to, defined, and measured, depending on the context (Table 1). Each pair of authors coded 2 samples of 10 papers independently. This resulted in no disagreement, the codes were straightforward to interpret.

**Table 1:** Distribution of the main codes (some studies performed multiple measurements).

Themes and codes			Studies, n (%)
Given definition			68 (100)
**Nature of given definition**			
	Operationalized	39 (58)	
	Synonym	22 (32)	
	Full definition	7 (10)	
**Cited reference for given definition**			
	Yes	13 (20)	
	No	55 (80)	
**Intervention domain**			
	Mental health	55 (80)	
	Health	12 (18)	
	Both	1 (2)	
**Goal of assessing acceptance**			
	Inform design	13 (19)	
	Evaluate a system	48 (71)	
	Review the literature	7 (10)	
**Acceptance measurement**			
	Customized survey	39 (54)	
	Standardized survey	13 (18)	
	Usage	7 (10)	
	Interviews (qualitative)	6 (9)	
	Focus groups (qualitative)	5 (7)	
	Adherence	1 (1)	
	Completion of task	1 (1)	
**Measurement timeline**			
	Preuse	16 (19)	
	Initial use	7 (10)	
	Sustained use	42 (61)	
	Unclear	1 (1)	
	No measurement	6 (9)	

### Collating, Summarizing, and Reporting the Findings

Definitions from the literature mentioned earlier distinguish between the different stages of the process of acceptance. We assessed whether the definitions found in our corpus also differentiate between the stages of acceptance and the different terms *acceptability*, *acceptance*, and *adoption*. We carried out 2 classifications of the papers, one with respect to the terminology used (ie, *acceptability*, *acceptance*, or *adoption*) and one following the measurement stage (ie, preuse, initial use, and sustained use). We then grouped papers with similar interpretations of technology acceptance and extracted the measurements used. Finally, we identified possible gaps and suggested ways to improve the exploration of acceptance in mHealth.

## Results

All 3 terms (acceptance, acceptability, and adoption) were used in the mHealth literature sample and were associated with various meanings and measurements.

### Definitions

**Figure 2:**
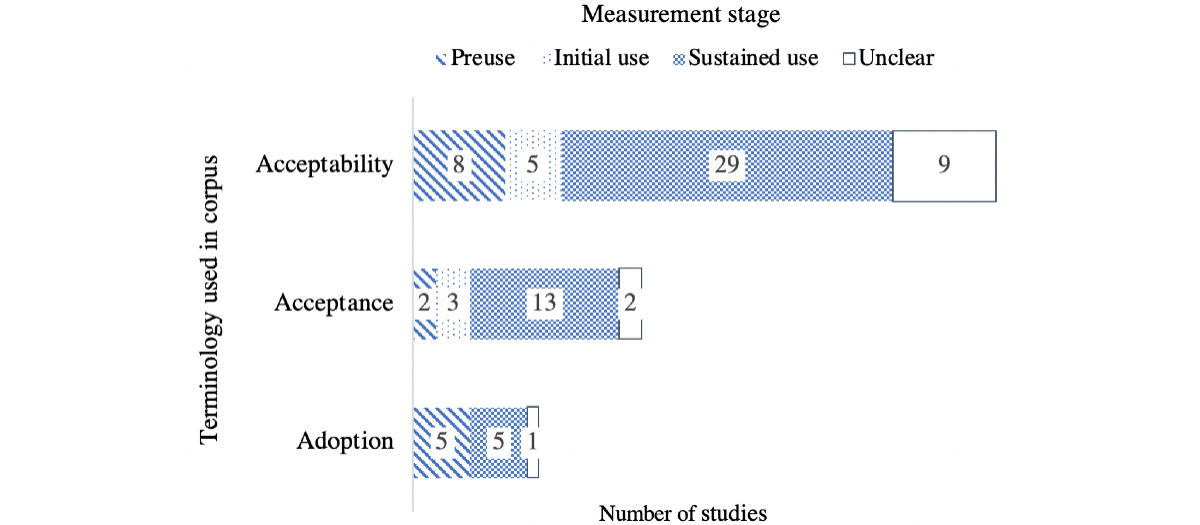
Comparison of the terminology used with a classification following the measurement stage (some papers employed multiple terms).

Classification of the papers reveals that one-fifth of them cited definitions from the literature. Two-third of the articles reported on a mental health intervention, which shows that the question of technology acceptance is particularly studied in this context. Figure 2 compares the terminology used in the papers with a classification following the measurement stage. A significant difference can be seen between the 2 distributions, marking the distinction between the 3 terms, but also their use in relation to the measurement timeline.

The majority of publications (n=51) referred to technology acceptability. Among these, a small number (n=8) addressed the preuse stage, sometimes equating acceptability to users' interest or willingness to use a system [46-50]. The other 34 papers referred to acceptability in the context of initial or sustained use.

Papers in the second group (n=20) referred to technology acceptance. Among these, 2 papers refer to it at preuse, and 13 in the context of sustained use.

The last part of the corpus (n=11) refers to technology adoption. Half of these papers focused on the sustained use stage and the other half on the preuse stage. Papers reported as unclear were those for which classification was not possible as the context of the study was ambiguous. Although the terminology used showed a focus on acceptability, looking at the corpus through the lens of the measurement timeline highlighted that more than two-third of the papers (n=47) explored the sustained use stage. This distribution suggested a trend in the corpus to employ the term *acceptability*, regardless of the context and stage of the user journey. Table 2 presents the range of interpretations for acceptability, acceptance, and adoption extracted from the corpus.

**Table 2:** Meanings associated with terminology (some studies referred to several concepts).

Terms and associated meanings		Occurrences [references], n
**Acceptability**		
	Perceived usefulness	14 [51-64]
	User satisfaction	11 [54,55,57,60,65-71]
	System usability	8 [48,57,67,72-76]
	User feedback	8 [52,53,61,73,75,77-79]
	Rate of recommendation	8 [51,53,54,61,62,73,79,80]
	Actual usage	8 [64,79,81-86]
	Perceived efficiency	6 [51,59,66,73,79,86]
	Perceived ease of use	5 [52,53,63,64,73]
	Intention to use	5 [46,47,50,64,87]
	User engagement	4 [58,79,81,88]
	User enjoyment	4 [51,58,64,79]
	Attitude toward using	2 [49,64]
	Quality of the system	1 [64]
**Acceptance**		
	Perceived usefulness	5 [64,89-92]
	Intention to use	5 [64,87,90,91,93]
	Actual usage	5 [64,85,90,91,94]
	User satisfaction	4 [71,89,93,95]
	Perceived ease of use	3 [64,90,91]
	Attitude toward using	3 [64,90,91]
	Perceived efficiency	2 [89,93]
	System usability	2 [76,96]
	Quality of the system	2 [64,93]
	User feedback	1 [97]
	User enjoyment	1 [64]
**Adoption**		
	Actual usage	7 [84-86,91,98-100]
	Intention to use	3 [91,101,102]
	Perceived usefulness	2 [63,91]
	Perceived ease of use	2 [63,91]
	Perceived efficiency	1 [86]
	Attitude toward using	1 [91]

The distribution shown in Table 2 reveals that acceptability was mainly understood as perceived usefulness (a concept from TAM) or user satisfaction. System usability, user feedback, and the other TAM constructs (perceived ease of use, attitude toward using, intention to use, and actual usage) were often mentioned. It also emerged that researchers may consider a perceived reduced stigma [49] and high similarity between the behavior of the technology and traditional health care [61] as markers for acceptability. Furthermore, one study explored sociocultural aspects of technology acceptability in developing regions, explaining that a system needs to take into account “the preferences and aspirations of individual service users and the cultures of their communities” [103]. A striking finding was that, out of 6 years of research, only 2 works explored mHealth for children [87,104], with Farooqui et al [104] alone studying children’s acceptance.

Fewer studies employed the term acceptance, associating it with constructs from the TAM—perhaps because the model itself uses the term acceptance.

TAM constructs were also found in the interpretations of adoption. Some of this work seeks to adapt or extend the TAM, such as Khatun et al [105], who developed the concept of *readiness to adopt* mHealth in developing countries. The authors argued that the TAM “does not consider the influence of human factors, the internal resources of the user or the external environmental and ecological factors” and proposed a model integrating TAM constructs and others more specific to rural and developing areas (access, sociodemographics, awareness of mHealth services, and trust).

Among the literature reviews present in the corpus, some associated acceptability or acceptance with feasibility [97,106]. Feasibility, as defined by the National Institute for Health Research glossary, relates to whether a study can be carried out. A feasibility study explores not only technical parameters but also human factors likely to be important for the conduct of the main study. Although a feasibility study may include the assessment of participants’ acceptance, it is not the case that all feasibility studies will do so.

A small number of studies (n=13) cited conceptual definitions from the literature. The TAM was cited in reference to the 3 concepts: acceptability [64,65], acceptance [64,90,91], and adoption [91,102,105].

However, some studies have highlighted the limitations of existing models. Zhu et al [102] argue that conceptual models should not regard mobile services as a generic concept but specifically address particular use cases (eg, mobile services for health monitoring). They present their own model for technology acceptance, combining TAM constructs with health-related concepts from the health belief model [107].

In the same vein, Povey et al [108] stated that existing models were not suitable for their study. The authors attempted to build a model for the acceptability of e-mental health for an indigenous population and acknowledged that the resulting model is similar to the HITAM [31].

Ammenwerth et al [109] argued that existing models such as the TAM and task technology fit failed to address the interaction between user and task. To bridge this gap, the authors developed and validated the fit between individuals, task, and technology (FITT) framework for information technology adoption in clinical environments. Only one study [85] then employed FITT to measure acceptance of the use of mobile apps among physical therapists. Finally, another study [98] cited Agarwal and Prasad [110], supporting the idea of 2 stages: initial adoption and long-term engagement.

These references to models and definitions within the literature show researchers’ willingness to reuse existing theories. However, the adaptations of these models and their inconsistent interpretation also reveal their limitations and the lack of a common framework to study technology acceptance.

### Measurement

We extracted measurements employed in the corpus to assess technology acceptance and classified them by type and temporality (Table 3).

**Table 3:** Measurements performed (some studies performed several measurements).

Measurement	Occurrences per stage		
	Preuse (11 studies), n	Initial use (7 studies), n	Sustained use (40 studies), n
Customized survey	7	2	28
Standardized survey	2	2	8
Focus groups (qualitative)	2	1	1
Interviews (qualitative)	1	1	4
Completion of task	0	1	1
Usage	0	0	6

Table 3 illustrates a strong preference for the use of surveys (73.0%, 49/67), independently of the timeline, with a prevalence of custom questionnaires (55.0%, 37/67). Almost all studies assessing system usability (which is a frequent interpretation of acceptability, see Table 2), made use of standardized surveys, which reflects the range of reliable tools available to evaluate this concept [72,74].

Other studies used existing surveys incorporating TAM constructs [64,91] or developed their own with elements from different models to fit their research better [102]. Owing to the need to assess technology acceptance in developing countries and remote areas, Chen et al [64] suggested that models found in the literature may need to be validated on a larger variety of populations.

The rest of the corpus measured technology acceptance through participant feedback (via custom surveys, focus groups, and interviews), adherence, usage, and rate of completion of tasks. Although this wide set of metrics contributes to expanding the number of assessment tools, it also impedes consistency within technology acceptance research.

The low number of measures applied at the pre and initial use stages also reveals that technology acceptance was rarely investigated at the design stage (Table 4).

**Table 4:** Measurement timeline of constructs (some studies measured several constructs).

Constructs (occurrences)	Measurement stage [references]		
	Preuse (n=13)	Initial use (n=7)	Sustained use (n=87)
Perceived usefulness (n=18)	[61]	[57,58]	[52-56,58-60,62-64,89-92]
User satisfaction (n=15)	—^a^	[57,65]	[54,55,60,62,66-71,89,93,95]
Intention to use (n=11)	[46,47,50,101,102,105]	—	[64,87,90,91,93]
System usability (n=10)	[48]	[57]	[55,67,72-76,96]
Rate of recommendation (n=10)	[61]	—	[53-55,62,73,79,80,104,111]
Actual usage (n=9)	—	—	[64,79,81,83,84,86,90,91,94]
User feedback (n=8)	[61,77]	[78]	[52,53,73,75,79]
Perceived ease of use (n=7)	—	—	[52,53,63,64,73,90,91]
Perceived efficiency (n=6)	—	—	[59,66,73,79,86,89,96]
Attitude toward use (n=4)	[49]		[64,90,91]
User enjoyment (n=4)	[58]	[58]	[64,79]
User engagement (n=3)	—	—	[79,81,88]
Quality of the system (n=2)	—	—	[64,93]

^a^No study measured the construct at that specific measurement stage.

Interestingly, although existing acceptance models provide sets of measurable constructs, researchers’ efforts have focused on perceived usefulness. Only the studies in Table 5 measured the full set of constructs contained in the technology acceptance models which they cite.

**Table 5:** Use of existing technology acceptance models.

Acceptance models used in the corpus	Measurement stage			Additional constructs
	Preuse	Initial use	Sustained use	
TAM^a^	[102]	—^b^	—	Perceived disease threat, perceived risk, initial trust, and technology anxiety
	—	—	[64]	System quality, social influence, perceived enjoyment, and smartphone experience
	—	—	[90]	Demographics (age, position at work, usage time of PDA^c^, and skill level of using a PDA)
	—	—	[91]	None
Information system success model	—	—	[93]	None

^a^TAM: technology acceptance model (Davis [30]).

^b^Model was not used at that measurement stage.

^c^PDA: personal digital assistant.

Table 5 shows that almost all studies that relied on the TAM added constructs to capture the influence of context-related factors on acceptance. Indeed, Zhu et al [102] followed the added variables approach described by Holden and Karsh [112] to evaluate the acceptance of their technology within the specific Chinese health care context. In addition to the TAM constructs, they used 4 context-related constructs taken from other studies. Similarly, Chen et al [64] used a questionnaire based on the TAM with additional constructs from other studies to fit the context of their application. Finally, Wang et al [90] showed the influence of certain demographic factors (age, position at work, usage time of the personal digital assistant [PDA], and skill level of using a PDA) on the TAM constructs (perceived usefulness and ease of use).

On the other hand, Ammenwerth et al [93] used a survey based on the information system success model [113] to evaluate acceptance postuse at 2 different points in time. The first survey assessed the 7 constructs from the model, whereas the second only assessed intention to use and net benefit. This could suggest that these constructs are seen as more stable or important, or that the existing constructs are not suitable for all study designs.

As expected from the analysis of definitions, these studies measured a wide variety of constructs. New questionnaires were developed, and researchers stressed the limitations of existing measurement frameworks. Without the validation of these new surveys, the comparison and replication of studies may be difficult. Thus, we argue that standardized ways to measure technology acceptance are needed to support objective and coherent assessments at the different stages of the user journey.

## Discussion

This scoping review analyzed the terminology used to refer to technology acceptance and extracted the different interpretations given and measurement methods employed.

### Terminology

The findings reveal that technology acceptance was mostly referred to as acceptability and, to a lesser extent, acceptance or adoption. A small part of the corpus converges toward the differentiation of these terms as distinct concepts, corresponding to stages (ie, preuse acceptability, initial and sustained use acceptance) or events (ie, point of adoption) in the user journey of technology acceptance. However, the rest of the corpus does not distinguish between these terms. One reason could be that the existing definitions arguing for that distinction were published separately; hence, providing a coherent and more precise terminology and set of definitions can aid researchers in communicating which concepts they are referring to.

### Definitions

The corpus provided a variety of interpretations of the concept of technology acceptance. Among these, some were based on constructs present in literature models such as the TAM [30], indicating that existing models do influence acceptance studies. Other definitions should be associated with the appropriate model constructs; for example, user satisfaction and feedback should refer to the TAM’s construct *attitude toward use*. Similarly, factors related to the sensitive nature of health care technologies (such as perceived stigma) should be linked to the appropriate constructs’ *subjective norm* (in the HITAM), *social influence* (in the pervasive technology acceptance model), or *sharing* (in the model by Cheung et al [34]). Thus, it is likely that the extent to which an individual is concerned by these risks will impact their acceptance of the technology. Following previous research [102,108], we argue that acceptance models should take into account the cultural and health context of end users.

### Models and Measurement

Although part of the corpus employed standardized tools, the majority of the studies used custom surveys. This aligns with the findings of Wozney et al [5] that the major part of their corpus used nonvalidated measures of acceptability. This could be because of a lack of validated tools to assess technology acceptance in the context of mHealth. Many researchers felt the need to design their own survey to have a measurement instrument adapted to the specific issues of their target population (eg, technology access and cost). Similarly, researchers who used existing acceptance models felt the need to add context-specific constructs. It would be interesting to see a community effort to validate new tools, create adapted tools for important contexts such as mHealth, and adapt existing models and questionnaires so that they embrace the changes in users’ acceptance as they use the technology. Finally, technology acceptance was rarely investigated at the design stage. This is unexpected as exploring acceptance issues could greatly inform design work [114] and reduce risks that an implemented technology is rejected or abandoned. On the use of machine learning in clinical contexts, Thieme et al [115] argue that collaborating with health care users at the design stage may increase the chances of acceptance of the technology. Existing acceptance models were only used once to measure acceptance at the preuse stage (Table 5). A reason for this might be that existing models do not target the design stage and are not adapted to measurement at the preuse stage.

### Technology Acceptance Lifecycle

**Figure 3:**
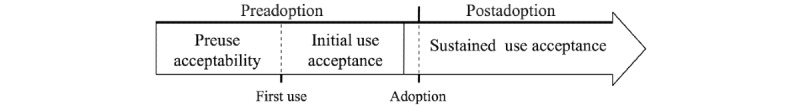
Proposed terminology for technology acceptance lifecycle.

In line with the existing literature definitions and informed by our analysis of the corpus, we argue for clearly distinguishing between the different stages of technology acceptance. We have seen in our analysis both a wide variety of interpretations of these concepts, and a range of measurement approaches applied across the lifecycle, mostly based on ad hoc tools. This variety creates ambiguities in the reporting and understanding of results and makes it difficult to draw conclusions on the acceptance of the systems studied. We believe that a better understanding of the process of technology acceptance would greatly benefit the community in terms of researchers articulating their findings with regard to the entire process. To contribute toward clarification of the measurement of technology acceptance, we propose the Technology Acceptance Lifecycle (TAL). The TAL consists of a timeline to anchor the definitions of technology acceptance within the overall process (Figure 3).

Our motivation is to highlight the evolving nature of technology acceptance across the different stages of the user journey with a technology. The two main stages of the TAL follow Karahanna et al [28] and Roger's interpretations [29] in favor of a distinction between the pre- and postadoption stages. With regard to the definitions of Distler et al [21], Martin et al [20], Garces et al [23], and Terrade et al [22], we argue for a distinction between the stages of acceptability (preuse) and acceptance (initial use) and propose the continuum acceptability-acceptance-sustained use. The TAL proposes a more explicit terminology, embedding temporality in the name of the different stages. The continuum then becomes preuse acceptability—initial use acceptance—sustained use acceptance. Finally, sometime during the sustained use stage, the user would reach the point of adoption of the technology. According to Rogers [29], a system can be considered adopted when users make full use of it. However, the literature does not specify the conditions for full use to be achieved. On the basis of the existing literature definitions, the TAL articulates the process of technology acceptance and its different stages across the user journey. We argue that acceptance at the initial and sustained use stages is likely to be impacted by factors related to the actual use of the technology and user engagement. Therefore, we suggest that the research community considers acceptance as a process (rather than a discrete measure) and adopts assessment approaches that take into account the temporal dimension and possible evolution of acceptance. We believe that the proposed TAL could help align the research field and provide researchers with a timeline that they can follow to assess technology acceptance and terminology to communicate their research clearly. Further research is needed to establish the influencing factors at the different stages of the process and to develop and validate measurement methods adapted to these stages.

### Limitations

Our search focused on the PubMed database and relied on the MeSH classification. We did not apply any time restriction and obtained a reasonably sized and highly relevant sample. However, a more exhaustive sample could be obtained by expanding the search to other terms.

### Conclusions

This review has identified the common interpretations and measurement approaches that have been used to assess the acceptance of mHealth technologies. To our knowledge, this is the first study to look at the terminology employed and examine the basis and consistency of the definitions employed in mHealth research. In addition, this review has described how researchers made use of existing models to measure mHealth acceptance and the lack of readily available assessment tools that are appropriate for specific study contexts and use at the design stage. This paper has uncovered the need for a common definition and measurement framework to address technology acceptance, particularly in the domains of health and mental health. A common set of definitions and more consistent approaches to measurement would support both developers of mHealth applications in addressing user acceptance of their systems and the communication and reporting of results in interdisciplinary studies of mHealth interventions. With the TAL, we propose a more explicit terminology and a representation of the process of technology acceptance throughout the user journey.

Our findings highlight the importance of better articulating the specific concepts highlighted in the TAL and developing appropriate measurement tools, ideally standardized, for each of these concepts. This perspective encourages developers to consider acceptance across the user journey and allows researchers to be more explicit about what they are investigating. Finally, efforts from the mHealth community are needed to adapt existing acceptance models for use in sensitive contexts such as mental health interventions.

## Abbreviations

FITT: fit between individuals, task, and technology
HCI: human-computer interaction
HITAM: health information technology acceptance model
MeSH: Medical Subject Headings
mHealth: mobile health
NASSS: nonadoption, abandonment, scale-up, spread, and sustainability framework
PDA: personal digital assistant
TAL: Technology Acceptance Lifecycle
TAM: technology acceptance model

## References

[1] Sanches P, Janson A, Karpashevich P, Nadal C, Qu C, Dauden RC, Umair M, Windlin C, Doherty G, Hook K, Sas C (2019). HCI and Affective Health: Taking Stock of a Decade of Studies and Charting Future Research Directions. Proceedings of the 2019 CHI Conference on Human Factors in Computing Systems.

[2] Coman A, Sas C (2016). A hybrid intervention for challenging the stigma of mental illness. Bulletin of the Transilvania University of Braşov, Series VII: Social Sciences and Law.

[3] Stowell E, Lyson M, Saksono H, Wurth R, Jimison H, Pavel M, Parker A (2018). Designing and Evaluating mHealth Interventions for Vulnerable Populations: A Systematic Review. Proceedings of the 2018 CHI Conference on Human Factors in Computing Systems.

[4] Sekhon M, Cartwright M, Francis JJ (2017). Acceptability of healthcare interventions: an overview of reviews and development of a theoretical framework. BMC Health Serv Res.

[5] Wozney L, McGrath PJ, Gehring ND, Bennett K, Huguet A, Hartling L, Dyson MP, Soleimani A, Newton AS (2018). eMental healthcare technologies for anxiety and depression in childhood and adolescence: systematic review of studies reporting implementation outcomes. JMIR Ment Health.

[6] Matthews M, Doherty G (2011). In the Mood: Engaging Teenagers in Psychotherapy Using Mobile Phones. Proceedings of the 2011 CHI Conference on Human Factors in Computing Systems.

[7] Yardley L, Spring BJ, Riper H, Morrison LG, Crane DH, Curtis K, Merchant GC, Naughton F, Blandford A (2016). Understanding and promoting effective engagement with digital behavior change interventions. Am J Prev Med.

[8] Qu C, Sas C, Doherty G (2019). Exploring and Designing for Memory Impairments in Depression. Proceedings of the 2019 CHI Conference on Human Factors in Computing Systems.

[9] Salovaara A, Hook K, Cheverst K, Twidale M, Chalmers M, Sas C (2011). Appropriation and creative use: linking user studies and design. CHI'11 Extended Abstracts on Human Factors in Computing Systems.

[10] Al-Youssef I (2015). Student acceptance and use of internet-based distance education in Saudi Electronic University (SEU): a mixed method study.

[11] Dillon A, Morris M (1996). User acceptance of new information technology: theories and models. Annual Review of Information Science and Technology.

[12] Fischer AJ, Dart EH, Leblanc H, Hartman KL, Steeves RO, Gresham FM (2016). An investigation of the acceptability of videoconferencing within a school-based behavioral consultation framework. Psychology in the Schools.

[13] Schade J, Schlag B (2003). Acceptability of urban transport pricing strategies. Transp Res Part F Traffic Psychol Behav.

[14] Adell E (2010). Acceptance of driver support systems. https://www.researchgate.net/publication/229049067_Acceptance_of_driver_support_systems.

[15] (2020). Acceptability. Cambridge Dictionary - Cambridge University Press.

[16] (2020). Acceptance. Cambridge Dictionary - Cambridge University Press.

[17] Proctor E, Silmere H, Raghavan R, Hovmand P, Aarons G, Bunger A, Griffey R, Hensley M (2011). Outcomes for implementation research: conceptual distinctions, measurement challenges, and research agenda. Administration and Policy in Mental Health and Mental Health Services Research.

[18] Forlizzi J, Battarbee K (2004). Understanding Experience in Interactive Systems. Proceedings of the 5th conference on Designing interactive systems: processes, practices, methods, and techniques.

[19] Hassenzahl M, Tractinsky N (2006). User experience - a research agenda. Behaviour & Information Technology.

[20] Martin N, Erhel S, Jamet E, Rouxel G (2015). What Links Between User Experience and Acceptability?. Proceedings of the 27th Conference on l'Interaction Homme-Machine.

[21] Distler V, Lallemand C, Bellet T (2018). Acceptability and Acceptance of Autonomous Mobility on Demand: The Impact of an Immersive Experience. Proceedings of the 2018 CHI Conference on Human Factors in Computing Systems.

[22] Terrade F, Pasquier H, Reerinck-Boulanger J, Guingouain G, Somat A (2009). [L'acceptabilité sociale: la prise en compte des déterminants sociaux dans l'analyse de l'acceptabilité des systèmes technologiques]. Le Travail Humain.

[23] Arbelaez Garces G, Rakotondranaivo A, Bonjour E (2016). An acceptability estimation and analysis methodology based on Bayesian networks. Int J Ind Ergon.

[24] Reerink-Boulanger J (2012). [Services technologiques intégrés dans l’habitat des personnes âgées: examen des déterminants individuels, sociaux et organisationnels de leur acceptabilité] (Doctoral dissertation). HAL Archives.

[25] Overdijk M, Van Diggelen W (2006). Technology appropriation in face-to-face collaborative learning. Proceedings of the European Conference on Technology Enhanced Learning.

[26] Bar F, Weber MS, Pisani F (2016). Mobile technology appropriation in a distant mirror: baroquization, creolization, and cannibalism. New Media Soc.

[27] Carroll J, Howard S, Peck J, Murphy J (2003). From adoption to use: the process of appropriating a mobile phone. Australasian J Inf Syst.

[28] Karahanna E, Straub DW, Chervany NL (1999). Information technology adoption across time: a cross-sectional comparison of pre-adoption and post-adoption beliefs. MIS Q.

[29] Rogers E (2010). Diffusion of Innovations.

[30] Davis F (1986). A technology acceptance model for empirically testing new end-user information systems: Theory and results.

[31] Kim J, Park H (2012). Development of a health information technology acceptance model using consumers' health behavior intention. J Med Internet Res.

[32] Connelly K (2007). On Developing a Technology Acceptance Model for Pervasive Computing. 9th International Conference on Ubiquitous Computing -Workshop of Ubiquitous System Evaluation (USE) New York, NY. In 9th International Conference on Ubiquitous Computing - Workshop of Ubiquitous System Evaluation.

[33] Dou K, Yu P, Deng N, Liu F, Guan Y, Li Z, Ji Y, Du N, Lu X, Duan H (2017). Patients' acceptance of smartphone health technology for chronic disease management: a theoretical model and empirical test. JMIR Mhealth Uhealth.

[34] Cheung ML, Chau KY, Lam MH, Tse G, Ho KY, Flint SW, Broom DR, Tso EK, Lee KY (2019). Examining consumers' adoption of wearable healthcare technology: the role of health attributes. Int J Environ Res Public Health.

[35] Doherty K, Doherty G (2019). Engagement in HCI. ACM Comput Surv.

[36] Karapanos E, Hassenzahl M, Martens J (2008). User Experience over Time. In CHI ’08 Extended Abstracts on Human Factors in Computing Systems.

[37] Karapanos E, Zimmerman J, Forlizzi J (2009). User Experience Over Time: an Initial Framework. Proceedings of the SIGCHI Conference on Human Factors in Computing Systems.

[38] Greenhalgh T, Wherton J, Papoutsi C, Lynch J, Hughes G, A'Court C, Hinder S, Fahy N, Procter R, Shaw S (2017). Beyond adoption: a new framework for theorizing and evaluating nonadoption, abandonment, and challenges to the scale-up, spread, and sustainability of health and care technologies. J Med Internet Res.

[39] Dworkin M, Panchal P, Jimenez A, Garofalo R, Haberer J, Wiebel W (2017). How acceptable is a wireless pill bottle that monitors and texts in response to missed doses: focus groups with young African American MSM living with HIV. Open Forum Infect Dis.

[40] Lawson-Body A, Willoughby L, Lawson-Body L, Tamandja EM (2018). Students’ acceptance of E-books: an application of UTAUT. J Comp Inf Syst.

[41] Hermes ED, Lyon AR, Schueller SM, Glass JE (2019). Measuring the implementation of behavioral intervention technologies: recharacterization of established outcomes. J Med Internet Res.

[42] Allen I, Seaman J (2008). Staying the Course: Online Education in the United States, 2008. Sloan Consortium.

[43] Otto L, Harst L, Schlieter H, Wollschlaeger B, Richter P, Timpel P (2018). Towards a unified understanding of ehealth and related terms-proposal of a consolidated terminological basis. Proceedings of the 11th International Joint Conference on Biomedical Engineering Systems and Technologies - Volume 5: HEALTHINF.

[44] Levac D, Colquhoun H, O'Brien KK (2010). Scoping studies: advancing the methodology. Implement Sci.

[45] Arksey H, O'Malley L (2005). Scoping studies: towards a methodological framework. Int J Soc Res Methodol.

[46] Sharpe JD, Zhou Z, Escobar-Viera CG, Morano JP, Lucero RJ, Ibañez GE, Hart M, Cook CL, Cook RL (2018). Interest in using mobile technology to help self-manage alcohol use among persons living with the human immunodeficiency virus: A Florida Cohort cross-sectional study. Subst Abus.

[47] Abelson JS, Symer M, Peters A, Charlson M, Yeo H (2017). Mobile health apps and recovery after surgery: what are patients willing to do?. Am J Surg.

[48] Gardner CL, Liu F, Fontelo P, Flanagan MC, Hoang A, Burke HB (2017). Assessing the usability by clinicians of VISION: a hierarchical display of patient-collected physiological information to clinicians. BMC Med Inform Decis Mak.

[49] Liu X, Wang R, Zhou D, Hong Z (2016). Feasibility and acceptability of smartphone applications for seizure self-management in China: questionnaire study among people with epilepsy. Epilepsy Behav.

[50] Peterson CM, Apolzan JW, Wright C, Martin CK (2016). Video chat technology to remotely quantify dietary, supplement and medication adherence in clinical trials. Br J Nutr.

[51] Helsel BC, Williams JE, Lawson K, Liang J, Markowitz J (2018). Telemedicine and mobile health technology are effective in the management of digestive diseases: a systematic review. Dig Dis Sci.

[52] Guo Y, Chen Y, Lane DA, Liu L, Wang Y, Lip GY (2017). Mobile health technology for atrial fibrillation management integrating decision support, education, and patient involvement: mAF app trial. Am J Med.

[53] Westergaard RP, Genz A, Panico K, Surkan PJ, Keruly J, Hutton HE, Chang LW, Kirk GD (2017). Acceptability of a mobile health intervention to enhance HIV care coordination for patients with substance use disorders. Addict Sci Clin Pract.

[54] Schlosser DA, Campellone TR, Truong B, Anguera JA, Vergani S, Vinogradov S, Arean P (2017). The feasibility, acceptability, and outcomes of PRIME-D: a novel mobile intervention treatment for depression. Depress Anxiety.

[55] Gordon JS, Armin J, Hingle MD, Giacobbi P, Cunningham JK, Johnson T, Abbate K, Howe CL, Roe DJ (2017). Development and evaluation of the See Me Smoke-Free multi-behavioral mhealth app for women smokers. Transl Behav Med.

[56] Hicks TA, Thomas SP, Wilson SM, Calhoun PS, Kuhn ER, Beckham JC (2017). A preliminary investigation of a relapse prevention mobile application to maintain smoking abstinence among individuals with posttraumatic stress disorder. J Dual Diagn.

[57] O'Brien KH, LeCloux M, Ross A, Gironda C, Wharff EA (2017). A pilot study of the acceptability and usability of a smartphone application intervention for suicidal adolescents and their parents. Arch Suicide Res.

[58] Rizvi SL, Hughes CD, Thomas MC (2016). The DBT coach mobile application as an adjunct to treatment for suicidal and self-injuring individuals with borderline personality disorder: a preliminary evaluation and challenges to client utilization. Psychol Serv.

[59] Miner A, Kuhn E, Hoffman JE, Owen JE, Ruzek JI, Taylor CB (2016). Feasibility, acceptability, and potential efficacy of the PTSD coach app: a pilot randomized controlled trial with community trauma survivors. Psychol Trauma.

[60] Sureshkumar K, Murthy G, Natarajan S, Naveen C, Goenka S, Kuper H (2016). Evaluation of the feasibility and acceptability of the 'care for stroke' intervention in India, a smartphone-enabled, carer-supported, educational intervention for management of disability following stroke. BMJ Open.

[61] Juarascio AS, Goldstein SP, Manasse SM, Forman EM, Butryn ML (2015). Perceptions of the feasibility and acceptability of a smartphone application for the treatment of binge eating disorders: qualitative feedback from a user population and clinicians. Int J Med Inform.

[62] Goldstein CM, Gathright EC, Dolansky MA, Gunstad J, Sterns A, Redle JD, Josephson R, Hughes JW (2014). Randomized controlled feasibility trial of two telemedicine medication reminder systems for older adults with heart failure. J Telemed Telecare.

[63] Hirst JE, Mackillop L, Loerup L, Kevat DA, Bartlett K, Gibson O, Kenworthy Y, Levy JC, Tarassenko L, Farmer A (2015). Acceptability and user satisfaction of a smartphone-based, interactive blood glucose management system in women with gestational diabetes mellitus. J Diabetes Sci Technol.

[64] Chen YS, Wong JE, Ayob AF, Othman NE, Poh BK (2017). Can Malaysian young adults report dietary intake using a food diary mobile application? A pilot study on acceptability and compliance. Nutrients.

[65] Brittain K, Kamp K, Cassandras C, Salaysay Z, Gómez-Márquez J (2018). A mobile app to increase informed decisions about colorectal cancer screening among African American and Caucasian women: a pilot study. Gastroenterol Nurs.

[66] Niendam TA, Tully LM, Iosif A, Kumar D, Nye KE, Denton JC, Zakskorn LN, Fedechko TL, Pierce KM (2018). Enhancing early psychosis treatment using smartphone technology: a longitudinal feasibility and validity study. J Psychiatr Res.

[67] Jacobson AE, Vesely SK, Haamid F, Christian-Rancy M, O'Brien SH (2018). Mobile application vs paper pictorial blood assessment chart to track menses in young women: a randomized cross-over design. J Pediatr Adolesc Gynecol.

[68] Eisenhauer CM, Hageman PA, Rowland S, Becker BJ, Barnason SA, Pullen CH (2017). Acceptability of mhealth technology for self-monitoring eating and activity among rural men. Public Health Nurs.

[69] Isetta V, Torres M, González K, Ruiz C, Dalmases M, Embid C, Navajas D, Farré R, Montserrat JM (2017). A new mhealth application to support treatment of sleep apnoea patients. J Telemed Telecare.

[70] Carter MC, Burley VJ, Nykjaer C, Cade JE (2013). Adherence to a smartphone application for weight loss compared to website and paper diary: pilot randomized controlled trial. J Med Internet Res.

[71] Patel S, Jacobus-Kantor L, Marshall L, Ritchie C, Kaplinski M, Khurana PS, Katz RJ (2013). Mobilizing your medications: an automated medication reminder application for mobile phones and hypertension medication adherence in a high-risk urban population. J Diabetes Sci Technol.

[72] Schnall R, Cho H, Mangone A, Pichon A, Jia H (2018). Mobile health technology for improving symptom management in low income persons living with HIV. AIDS Behav.

[73] Santo K, Chow CK, Thiagalingam A, Rogers K, Chalmers J, Redfern J (2017). MEDication reminder APPs to improve medication adherence in coronary heart disease (MedApp-CHD) study: a randomised controlled trial protocol. BMJ Open.

[74] Crosby LE, Ware RE, Goldstein A, Walton A, Joffe NE, Vogel C, Britto MT (2017). Development and evaluation of iManage: a self-management app co-designed by adolescents with sickle cell disease. Pediatr Blood Cancer.

[75] Hartzler AL, Venkatakrishnan A, Mohan S, Silva M, Lozano P, Ralston JD, Ludman E, Rosenberg D, Newton KM, Nelson L, Pirolli P (2016). Acceptability of a team-based mobile health (mhealth) application for lifestyle self-management in individuals with chronic illnesses. Conf Proc IEEE Eng Med Biol Soc.

[76] Sarzynski E, Decker B, Thul A, Weismantel D, Melaragni R, Cholakis E, Tewari M, Beckholt K, Zaroukian M, Kennedy AC, Given C (2017). Beta testing a novel smartphone application to improve medication adherence. Telemed J E Health.

[77] Radhakrishnan K, Toprac P, O'Hair M, Bias R, Kim MT, Bradley P, Mackert M (2016). Interactive digital e-health game for heart failure self-management: a feasibility study. Games Health J.

[78] Ahern DK, Parker D, Eaton C, Rafferty C, Wroblewski J, Goldman R (2016). Patient-facing technology for identification of COPD in primary care. J Innov Health Inform.

[79] Simmons ES, Paul R, Shic F (2016). Brief report: a mobile application to treat prosodic deficits in autism spectrum disorder and other communication impairments: a pilot study. J Autism Dev Disord.

[80] Bucci S, Barrowclough C, Ainsworth J, Machin M, Morris R, Berry K, Emsley R, Lewis S, Edge D, Buchan I, Haddock G (2018). Actissist: proof-of-concept trial of a theory-driven digital intervention for psychosis. Schizophr Bull.

[81] Muroff J, Robinson W, Chassler D, López LM, Gaitan E, Lundgren L, Guauque C, Dargon-Hart S, Stewart E, Dejesus D, Johnson K, Pe-Romashko K, Gustafson DH (2017). Use of a smartphone recovery tool for Latinos with co-occurring alcohol and other drug disorders and mental disorders. J Dual Diagn.

[82] Hickey AM, Freedson PS (2016). Utility of consumer physical activity trackers as an intervention tool in cardiovascular disease prevention and treatment. Prog Cardiovasc Dis.

[83] Forman DE, LaFond K, Panch T, Allsup K, Manning K, Sattelmair J (2014). Utility and efficacy of a smartphone application to enhance the learning and behavior goals of traditional cardiac rehabilitation: a feasibility study. J Cardiopulm Rehabil Prev.

[84] Ofili EO, Pemu PE, Quarshie A, Mensah EA, Rollins L, Ojutalayo F, McCaslin A, Clair BS (2018). Democratizing discovery health with N=ME. Trans Am Clin Climatol Assoc.

[85] Noblin A, Shettian M, Cortelyou-Ward K, Schack Dugre J (2017). Exploring physical therapists' perceptions of mobile application usage utilizing the FITT framework. Inform Health Soc Care.

[86] Yasini M, Marchand G (2016). Adoption and use of a mobile health application in older adults for cognitive stimulation. Stud Health Technol Inform.

[87] Ginsburg AS, Tawiah Agyemang C, Ambler G, Delarosa J, Brunette W, Levari S, Larson C, Sundt M, Newton S, Borriello G, Anderson R (2016). mPneumonia, an Innovation for Diagnosing and Treating Childhood Pneumonia in Low-Resource Settings: A Feasibility, Usability and Acceptability Study in Ghana. PLoS One.

[88] Moore RC, Kaufmann CN, Rooney AS, Moore DJ, Eyler LT, Granholm E, Woods SP, Swendsen J, Heaton RK, Scott JC, Depp CA (2017). Feasibility and acceptability of ecological momentary assessment of daily functioning among older adults with HIV. Am J Geriatr Psychiatry.

[89] Rico TM, dos Santos MK, Fernandes VP, Madruga SW, Noguez PT, Barcelos CR, Santin MM, Petrarca CR, Dumith SC (2017). Text messaging (SMS) helping cancer care in patients undergoing chemotherapy treatment: a pilot study. J Med Syst.

[90] Wang Y, Xiao Q, Sun L, Wu Y (2016). Chinese nurses' acceptance of PDA: a cross-sectional survey using a technology acceptance model. Stud Health Technol Inform.

[91] DeVito DA, Song M, Myers B, Hawkins RP, Aubrecht J, Begey A, Connolly M, Li R, Pilewski JM, Bermudez CA, Dew MA (2013). Clinical trials of health information technology interventions intended for patient use: unique issues and considerations. Clin Trials.

[92] Mertens A, Brandl C, Miron-Shatz T, Schlick C, Neumann T, Kribben A, Meister S, Diamantidis CJ, Albrecht U, Horn P, Becker S (2016). A mobile application improves therapy-adherence rates in elderly patients undergoing rehabilitation: a crossover design study comparing documentation via iPad with paper-based control. Medicine (Baltimore).

[93] Ammenwerth E, Woess S, Baumgartner C, Fetz B, van der Heidt A, Kastner P, Modre-Osprian R, Welte S, Poelzl G (2015). Evaluation of an integrated telemonitoring surveillance system in patients with coronary heart disease. Methods Inf Med.

[94] Becker S, Brandl C, Meister S, Nagel E, Miron-Shatz T, Mitchell A, Kribben A, Albrecht U, Mertens A (2015). Demographic and health related data of users of a mobile application to support drug adherence is associated with usage duration and intensity. PLoS One.

[95] Wu X, Oliveria SA, Yagerman S, Chen L, DeFazio J, Braun R, Marghoob AA (2015). Feasibility and efficacy of patient-initiated mobile teledermoscopy for short-term monitoring of clinically atypical nevi. JAMA Dermatol.

[96] Fallah M, Yasini M (2017). A medication reminder mobile app: does it work for different age ranges. Stud Health Technol Inform.

[97] Bashshur RL, Howell JD, Krupinski EA, Harms KM, Bashshur N, Doarn CR (2016). The empirical foundations of telemedicine interventions in primary care. Telemed J E Health.

[98] Lee K, Kwon H, Lee B, Lee G, Lee JH, Park YR, Shin S (2018). Effect of self-monitoring on long-term patient engagement with mobile health applications. PLoS One.

[99] Lobelo F, Kelli HM, Tejedor SC, Pratt M, McConnell MV, Martin SS, Welk GJ (2016). The wild wild west: a framework to integrate mhealth software applications and wearables to support physical activity assessment, counseling and interventions for cardiovascular disease risk reduction. Prog Cardiovasc Dis.

[100] Schulte M, Liang D, Wu F, Lan Y, Tsay W, Du J, Zhao M, Li X, Hser Y (2016). A smartphone application supporting recovery from heroin addiction: perspectives of patients and providers in China, Taiwan, and the USA. J Neuroimmune Pharmacol.

[101] Giménez-Pérez G, Recasens A, Simó O, Aguas T, Suárez A, Vila M, Castells I (2016). Use of communication technologies by people with type 1 diabetes in the social networking era. A chance for improvement. Prim Care Diabetes.

[102] Zhu Z, Liu Y, Che X, Chen X (2018). Moderating factors influencing adoption of a mobile chronic disease management system in China. Inform Health Soc Care.

[103] Peiris D, Praveen D, Johnson C, Mogulluru K (2014). Use of mhealth systems and tools for non-communicable diseases in low- and middle-income countries: a systematic review. J Cardiovasc Transl Res.

[104] Farooqui N, Phillips G, Barrett C, Stukus D (2015). Acceptability of an interactive asthma management mobile health application for children and adolescents. Ann Allergy Asthma Immunol.

[105] Khatun F, Heywood AE, Ray PK, Hanifi SM, Bhuiya A, Liaw S (2015). Determinants of readiness to adopt mHealth in a rural community of Bangladesh. Int J Med Inform.

[106] Jongbloed K, Parmar S, van der Kop M, Spittal PM, Lester RT (2015). Recent evidence for emerging digital technologies to support global HIV engagement in care. Curr HIV/AIDS Rep.

[107] Rosenstock IM (1974). The health belief model and preventive health behavior. Health Educ Monogr.

[108] Povey J, Mills PP, Dingwall KM, Lowell A, Singer J, Rotumah D, Bennett-Levy J, Nagel T (2016). Acceptability of mental health apps for aboriginal and Torres strait islander Australians: a qualitative study. J Med Internet Res.

[109] Ammenwerth E, Iller C, Mahler C (2006). IT-adoption and the interaction of task, technology and individuals: a fit framework and a case study. BMC Med Inform Decis Mak.

[110] Agarwal R, Prasad J (1997). The role of innovation characteristics and perceived voluntariness in the acceptance of information technologies. Decis Sci.

[111] Hochstenbach LM, Zwakhalen SM, Courtens AM, van Kleef M, de Witte LP (2016). Feasibility of a mobile and web-based intervention to support self-management in outpatients with cancer pain. Eur J Oncol Nurs.

[112] Holden RJ, Karsh B (2010). The technology acceptance model: its past and its future in health care. J Biomed Inform.

[113] Delone W, McLean ER (2014). The DeLone and McLean model of information systems success: a ten-year update. J Manag Inform Syst.

[114] Matthews M, Voida S, Abdullah S, Doherty G, Choudhury T, Im S, Gay G (2015). In Situ Design for Mental Illness: Considering the Pathology of Bipolar Disorder in mHealth Design. Proceedings of the 17th International Conference on Human-Computer Interaction with Mobile Devices and Services.

[115] Thieme A, Belgrave D, Doherty G (2020). Machine learning in mental health: a systematic review of the HCI literature to support effective ML system design. ACM Trans Comput Hum Interact.

